# Intergenerational Social Connections Enhance Depression Treatment Access for Older Adults With Self-Neglect

**DOI:** 10.1093/geroni/igaf122.1991

**Published:** 2025-12-31

**Authors:** Jason Burnett, Jordan Brousard, Melba Hernandez-Tejada, Ron Acierno

**Affiliations:** UTHealth Houston, Houston, Texas, United States; UTHealth Houston, Houston, Texas, United States; UTHealth Houston, Houston, Texas, United States; The University of Texas Health Science Center, Houston, Texas, United States

## Abstract

Self-neglect (SN) is the most reported issue to Adult Protective Services (APS) nationwide, linked to high morbidity and mortality. An estimated 50-60% of individuals with SN also suffer from untreated depression, which impairs self-management of chronic conditions. While treating depression could improve self-care, many older adults with SN decline APS-offered services, including mental health referrals. Increasing referral acceptance is crucial for improving outcomes. Social disconnection contributes to both depression and healthcare avoidance. Our study explored whether fostering meaningful social connections could increase acceptance of depression treatment referrals. We developed an eight-week intergenerational phone-calling program pairing health professional students with APS SN clients. Students screened participants weekly using the PHQ-2, offering referrals if depression was detected in two consecutive weeks. Preliminary data show that 77% of APS SN clients screened positive for depression at baseline. Among 74 pairings, 54% qualified for a referral, 98% were offered a referral, and 80% agreed to be referred and receive a contact from a treatment center and were referred. These findings strongly support intergenerational connections as a supportive strategy demonstrating that newly developed empathy-based social connections can enhance mental health referral acceptance and improve treatment access for older adults living with SN.

